# Supplementary material for: Distribution of rhizosphere fungi of Kobresia humilis on the Qinghai-Tibet Plateau
Source: PeerJ. 2024 Feb 20;12:e16620. doi: 10.7717/peerj.16620 (PMC10885805; doi:10.7717/peerj.16620)
Supplement: Supplemental Information 2 [file peerj-12-16620-s002.docx]

Figure S1. Effective tags information of root and rhizosphere soil in *K.humilis*





Figure S2. OTU information of root and rhizosphere soil in *K.humilis*


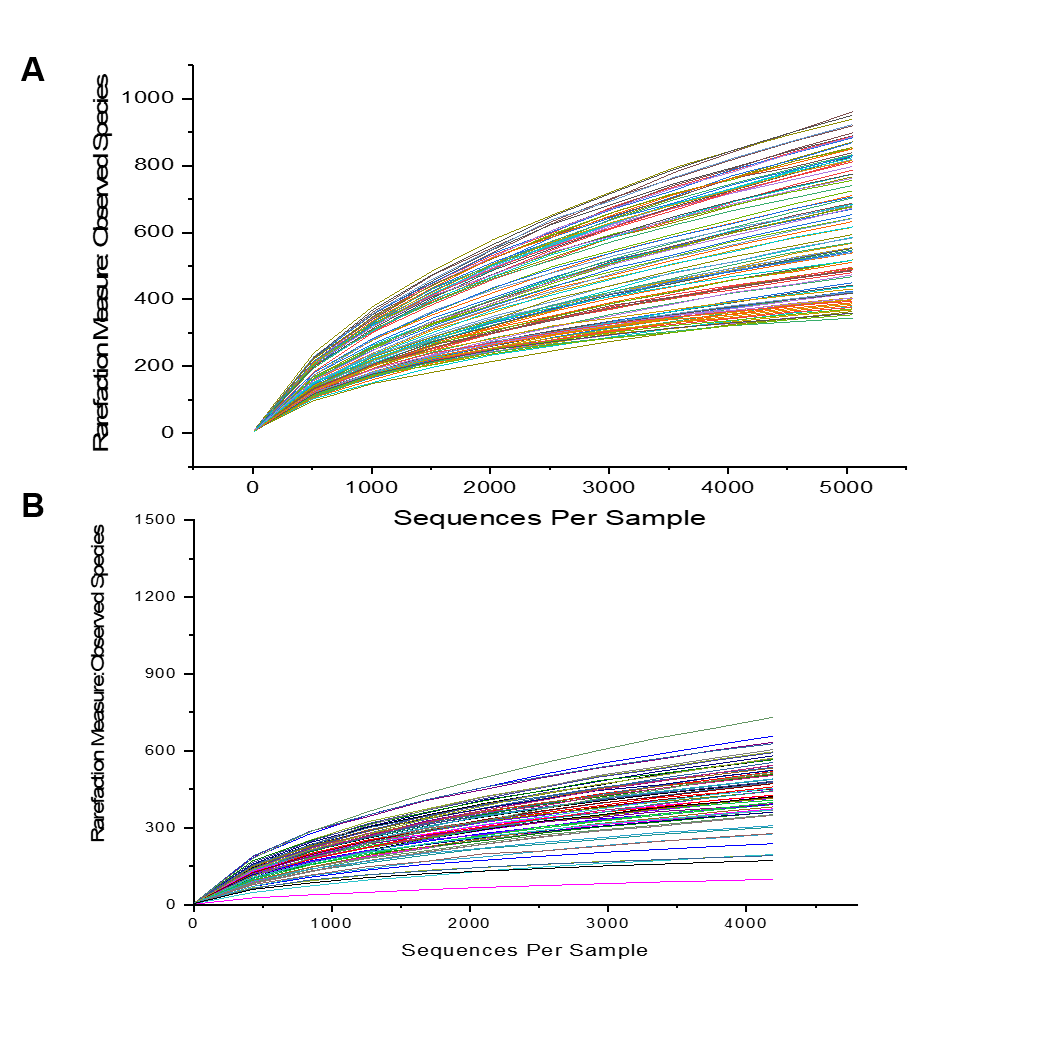


Figure S3. Rarefaction curves of fungal community in *K.humilis.* A Rarefaction curves of root. B Rarefaction curves of rhizosphere soil.


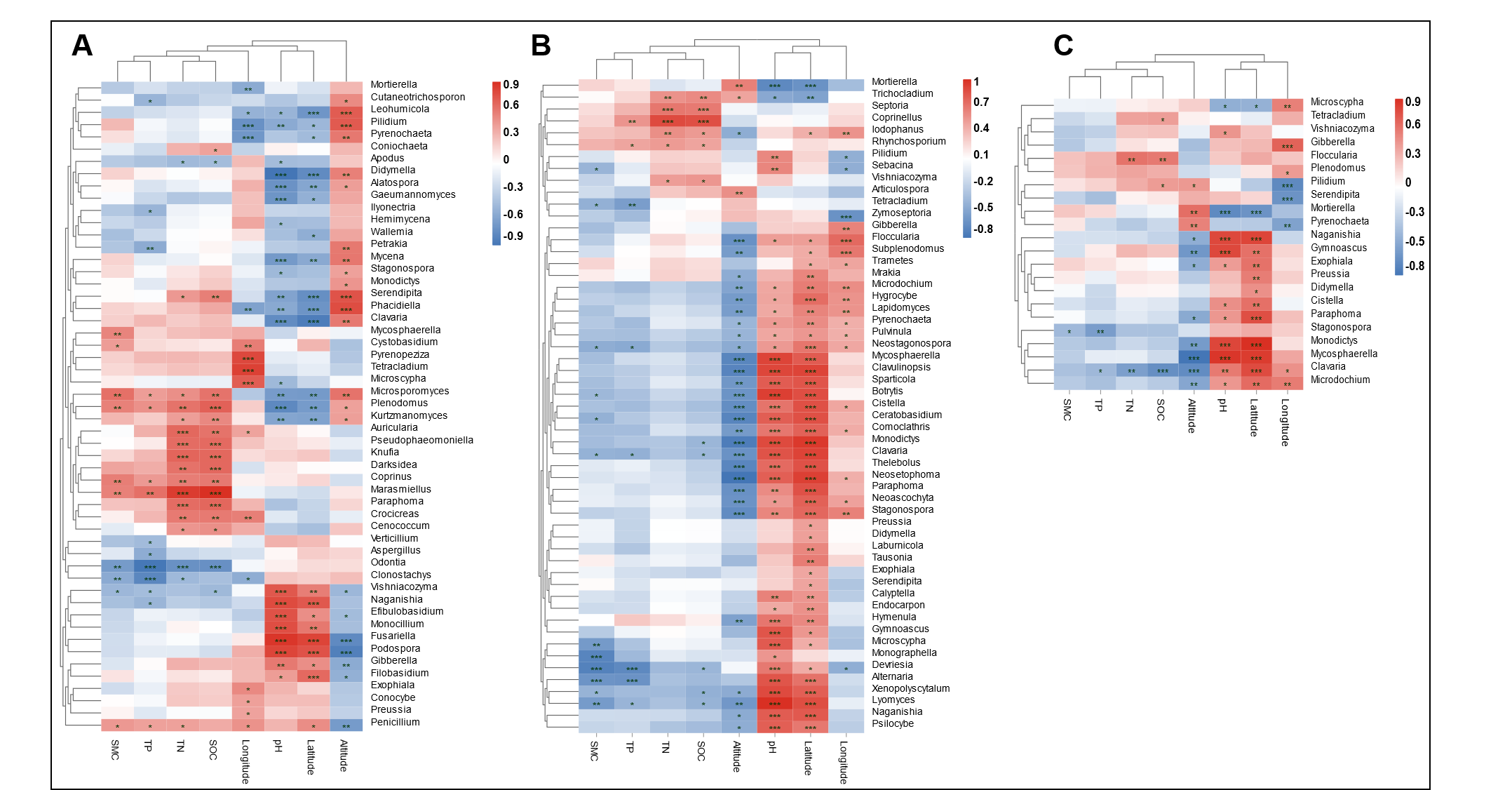


Figure S4. Heatmap diagram of the correlation between environmental factors and fungal community structure based on the core genus level at root, rhizosphere soil and the shared core genera among root and rhizosphere soil. A Heatmap diagram of the correlation between environmental factors and fungal community structure in root. B Heatmap diagram of the correlation between environmental factors and fungal community structure in rhizosphere soil. C Heatmap diagram of the correlation between environmental factors and fungal community structure based on the shared core genera among root and rhizosphere soil. R values are shown in different colors in the figure. P-values less than 0.05 are marked with ∗ (∗0.01 < P ≤ 0.05, ∗∗0.001 < P ≤ 0.01,∗∗∗P ≤ 0.001).
